# Supplementary material for: The Role of Histo-Blood Group Antigens and Microbiota in Human Norovirus Replication in Zebrafish Larvae
Source: Microbiol Spectr. 2022 Oct 31;10(6):e03157-22. doi: 10.1128/spectrum.03157-22 (PMC9769672; doi:10.1128/spectrum.03157-22)
Supplement: Supplemental file 1 — Fig. S1 to S5. Download spectrum.03157-22-s0001.pdf, PDF file, 0.6 MB [file spectrum.03157-22-s0001.pdf]

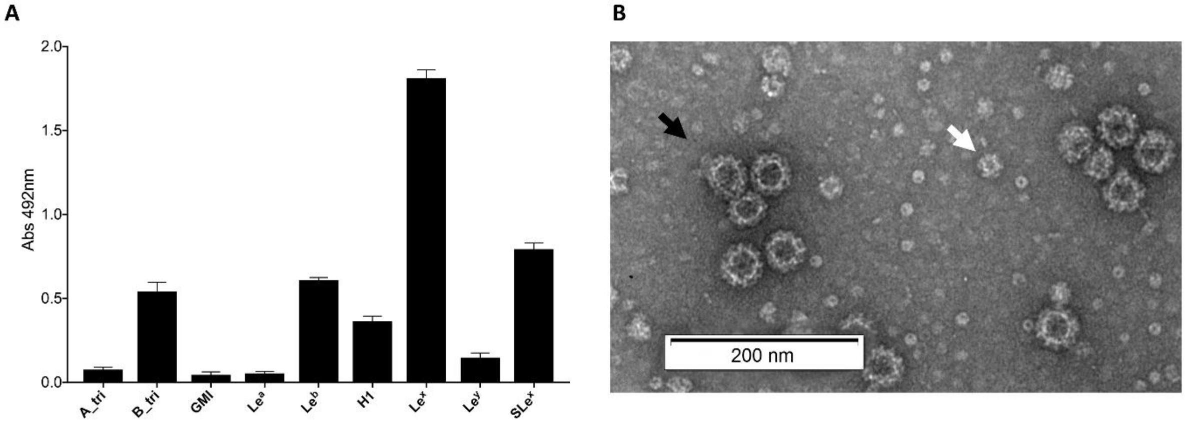

Supplementary figure 1: HuNoV GII.4 VLPs efficiently bind Le^x (A) ELISA-like binding assay of GII.4 2012 Sydney VLPs to a panel of sugar antigens conjugated to human serum albumin. GII.4 2012 Sydney VLPs were assayed to blood group A and B trisaccharides (A_Tri and B_tri respectively), GMI ganglioside (GMI), Lewis a (Le^a), Lewis b (Le^b), H-Type 1 (H1), Lewis X (Le^X), Lewis Y (Le^Y) and sialyl-Lewis X (SLe^X). **(B)** Transmission electron microscopy image showing negatively stained GII.4 2012 Sydney VLPs. The black arrow indicates a 40 nm putative T3 symmetry VLP. The white arrow points to a 23 nm putative T1 symmetry VLP.

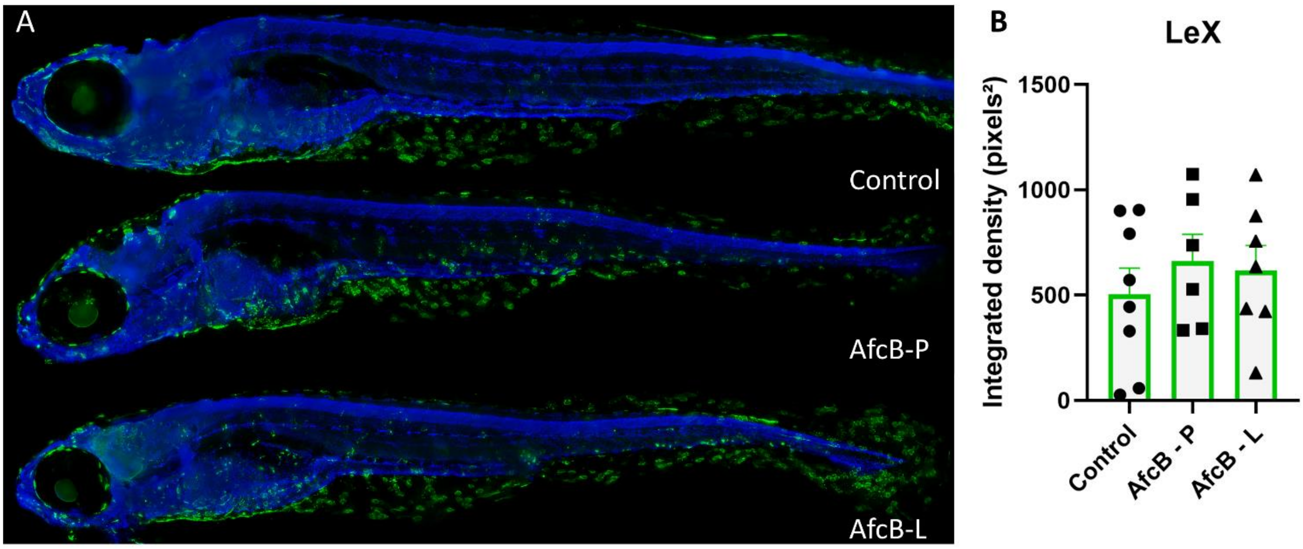

Supplementary figure 2: AfcB injection does not efficiently cleave LeX (A) Whole-mount immunohistochemistry staining in 5 dpf larvae of LeX after treatment with an α-1,3-fucosidase (AfcB). AfcB was injected at 3 dpf in the pericardial sac (P) or developing lumen (L). **(B)** LeX expression quantified using ImageJ. Integrated density was determined in an ROI comprising the whole intestinal tract.

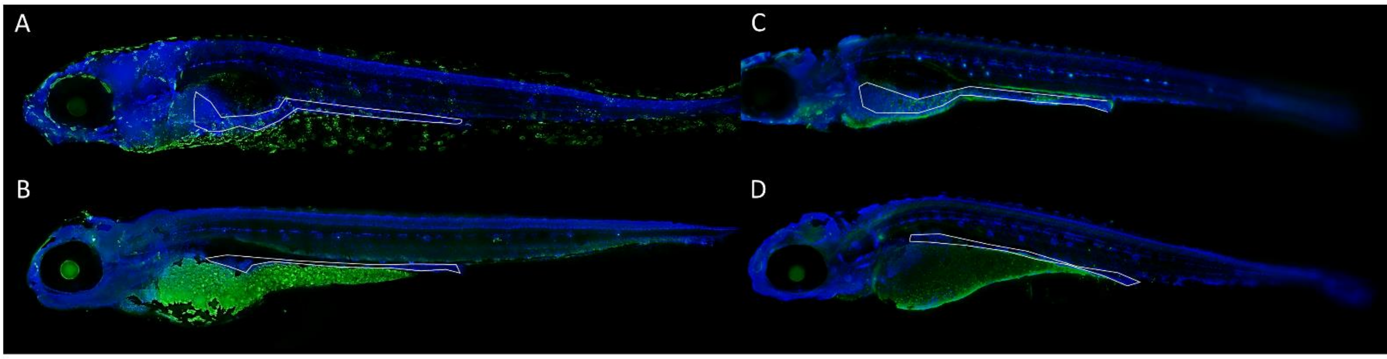

Supplementary figure 3: **Treatment with 2F-F successfully inhibits fucose expression. (A,B)** 5 dpf zebrafish larvae stained with anti-LeX antibody and counterstained with Hoechst 33342 (**A**) untreated, (**B**) treated with 500 µM 2F-F. **(C,D)** 5 dpf zebrafish larvae stained with FITC-AAL and counterstained with Hoechst 33342 **(C)** untreated, **(D)** treated with 500 µM 2F-F. Region defined by white line is the region of interest in the intestinal tract where Le^x/core fucose signal is quantified.

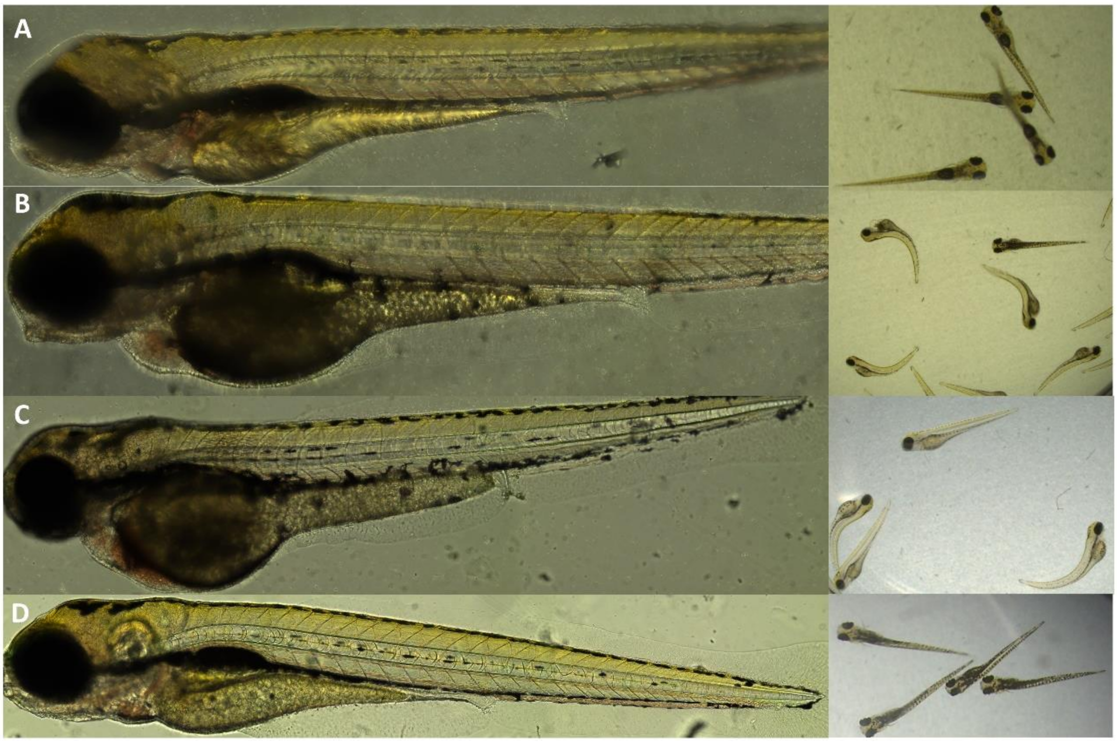

Supplementary figure 4: Developmental toxicity of 2F-Fucose in zebrafish larvae at 96 hpf. (A) Non-treated control larvae **(B)** 300 µM 2F-Fucose **(C)** 500 µM 2F-Fucose **(D)** 500 µM 3Fax-Neu5Ac

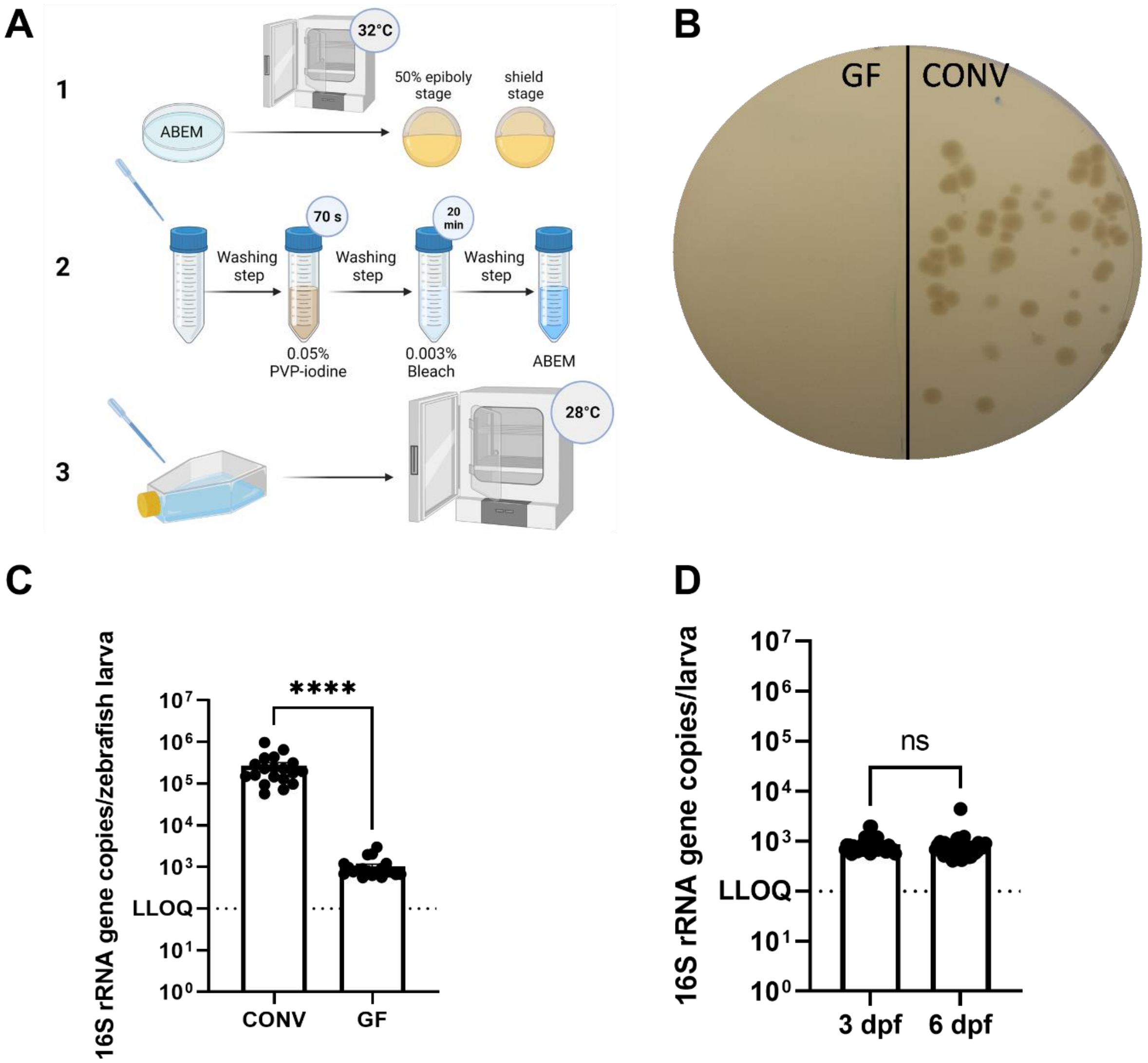

Supplementary figure 5: Generation and monitoring of germ-free zebrafish larvae (A) Schematic representation of the protocol to generate GF zebrafish larvae. Figure created with Bio Render **(B)** Bacterial growth from inoculation of 1 µL swimming water or 1 µL of homogenized zebrafish larvae on tryptic soy agar plates at 37°C. **(C)** Detection of 16S rRNA gene copies in CONV and GF zebrafish larvae. **(D)** Difference in 16S rRNA copy number/zebrafish larva between start (3 dpf) and end (6dpf/3 dpi) of the experiment. For graph C-D, ten GF zebrafish larvae were harvested, and bacterial load was determined by qPCR. The graphs represent the number of 16S rRNA gene copies/zebrafish larva whereby the bars represent the mean values ± SEM with 10^2 gene copies as LLOQ. Mann-Whitney tests were performed to detect significant differences.
